# A comparison of the influencing factors of chronic pain and quality of life between older Koreans and Korean–Americans with chronic pain: a correlational study

**DOI:** 10.1007/s11136-021-02983-2

**Published:** 2021-08-30

**Authors:** Sun Ju Chang, Hee Jun Kim, Hee-Soon Juon, Hyunjeong Park, Seung Woo Choi, Kyung-eun Lee, Hyunju Ryu

**Affiliations:** 1 College of Nursing and The Research Institute of Nursing Science, Seoul National University, Daehak-ro 103, Jongro-gu, Seoul, 406-799 Republic of Korea; 2 College of Nursing and Research Institute of Nursing Science, Ajou University, 206 World cup-ro, Yeongtong-gu, Suwon-si, Gyeonggi-do Republic of Korea; 3 Department of Medical Oncology, Thomas Jefferson University, Benjamin Franklin House, 834 Chestnut Street, Philadelphia, PA 19107 USA; 4 Department of Nursing, Towson University, 8000 York Road, Towson, MD 21252 USA; 5 Department of Nursing, College of Medicine, Inha University, 100 Inha-ro Bldg 5S-322, Michuhol-gu, Incheon, Republic of Korea; 6 College of Nursing, Seoul National University, Daehak-ro 103, Jongro-gu, Seoul, 406-799 Republic of Korea

**Keywords:** Chronic pain, Quality of life, Culture, Structural equation modeling

## Abstract

**Background:**

Chronic pain is one of the most common health problems for older adults worldwide and is likely to result in lower quality of life. Living in a different culture may also influence chronic pain and quality of life in older adults. The purpose of this study was to explore how multifaceted elements affect chronic pain and quality of life in older Koreans living in Korea and in older Korean–Americans (KAs) living in the USA.

**Methods:**

We conducted a secondary data analysis of data from 270 adults aged 65 years or over (138 Koreans and 132 KAs). We compared the effects of multifaceted elements on pain and quality of life by testing structural equation models (SEMs) for each group, using a maximum likelihood estimation and bootstrapping.

**Results:**

SEMs for both Korean and KAs showed that age and depressive symptoms directly affected quality of life. The number of comorbidities and depressive symptoms had mediating effects on quality of life through chronic pain in both groups. In older Koreans only, perceived financial status directly affected quality of life. In older KAs only, sleep quality indirectly affected quality of life through chronic pain.

**Conclusion:**

The data showed that multimorbidity and depressive symptoms play critical roles for explaining chronic pain in older Koreans and KAs and ultimately negatively influence quality of life. Future intervention program to improve quality of life in older adults with chronic pain should consider the different cultural aspects affecting quality of life for Koreans and KAs.

## Introduction

Persistent, recurrent chronic pain affects 39–70% of older adults worldwide [1–4] and negatively impacts their daily lives not only by causing discomfort, but also by limiting their activities, contributing to loneliness and social isolation. Chronic pain also influences older adults by costing them time and money to manage their pain and other health care needs [1, 5]. Older adults with chronic pain often also have chronic diseases, including musculoskeletal disorders (e.g., arthritis), endocrine disorders (e.g., diabetes), and cancers [6, 7], that further affect their well-being and complicate their health care needs. Therefore, older adults with chronic pain are likely to experience lower quality of life than those without chronic pain [8].

A biopsychosocial approach to pain is a well-known theoretical framework for understanding multi-dimensional aspects of pain [9]. A range of biological, psychological, and social factors can affect how people perceive pain and aging. For example, age [10], comorbidity [11], depressive symptoms and sleep quality [12], and economic status [13] have been reported as significant factors influencing chronic pain among elderly. In addition, the culture in which people live and other cultural aspects are pivotal to understanding chronic pain in older adults [14, 15]. Immigrant older adults may be particularly vulnerable to chronic pain because they are confronted with the cultural barriers and acculturation [15, 16]. Acculturation is a complex concept and can be defined as a process that occurs when an individual contacts two cultures and experiences substantial change in the culture [17]. Although previous studies have reported an association between acculturation and chronic pain, the results are somewhat sparse by age group; a relatively high level of acculturation was found among young immigrants [17, 18], and more acculturated immigrants had a higher prevalence of chronic pain [18]. Among elderly immigrants, a relatively low level of acculturation was reported [19], and acculturation did not directly correlate with somatization [20], which is often associated with pain complaints.

**Fig. 1:**
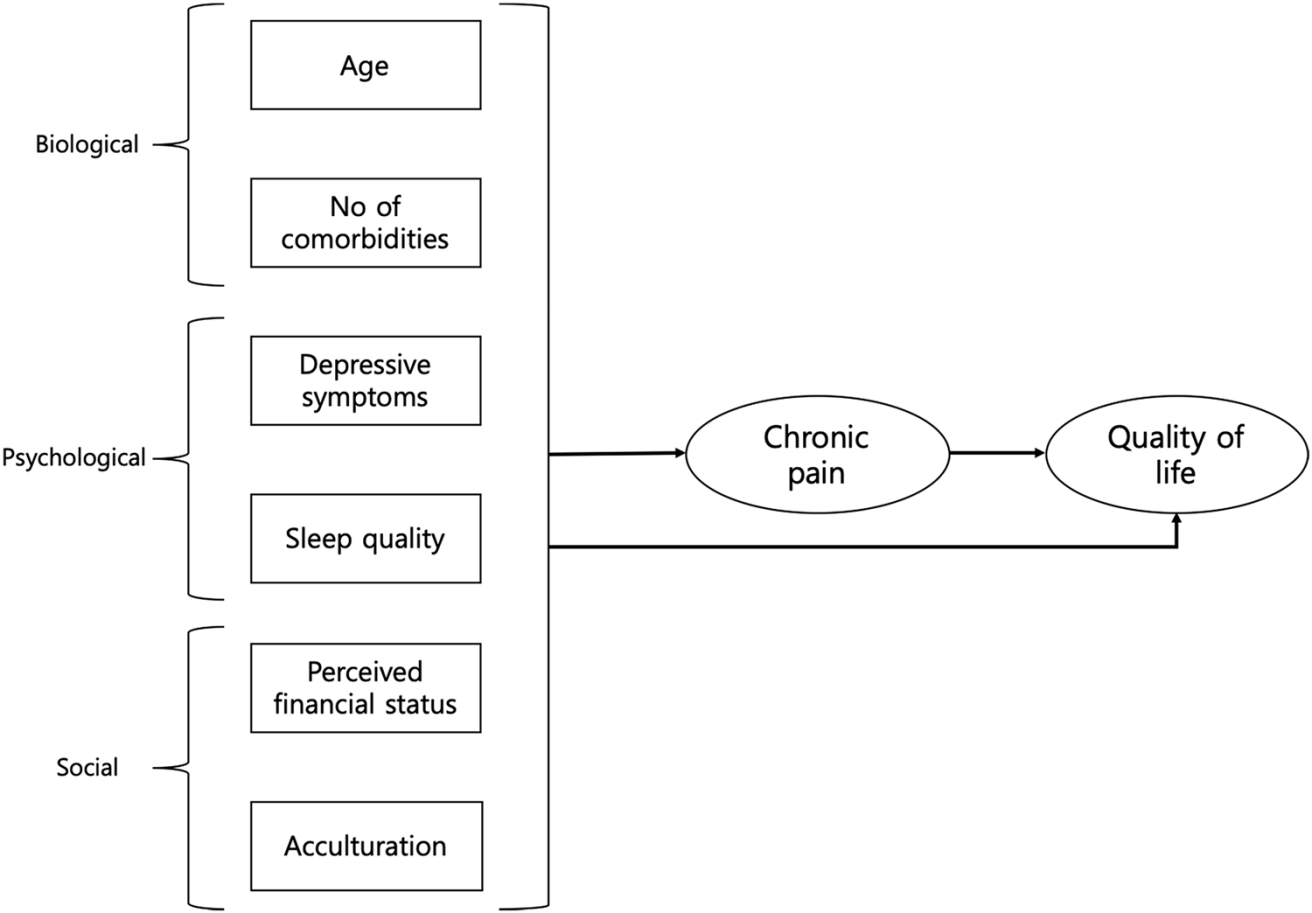
Research framework of this study

To better understand chronic pain and improve quality of life in older adults with chronic pain, a comprehensive approach that considers multifaceted elements affecting pain and quality of life should precede within a sociocultural context. In this study, we aimed to explore how multifaceted elements, including biological (age, the number of comorbidities), psychological (depressive symptoms, sleep quality), and social (perceived financial status, acculturation) aspects, affect chronic pain and quality of life in older adults with who have chronic pain and the same ethnicity, but who are living in different cultures (Fig. 1). We developed and compared two structural equation models which contained the paths from multifaceted elements to chronic pain and quality of life for older Korean–Americans (KAs) living in the USA and Koreans living in Korea. The hypothetical research framework is illustrated in Fig. 1.

## Methods

### Design

This study was a secondary data analysis of cross-sectional data from a previous study for exploring intra-ethnic differences in chronic pain and associated factors [11]. In order to compare the effects of multifaceted elements on pain and quality of life among older Koreans and KAs with chronic pain, we used a model testing correlation design.

### Participants

A total of 270 older adults (138 for Koreans living in Korea; 132 for KAs living in the USA) participated in the original study. Older adults (≥ 65 years of age) with chronic pain for the last 3 months, no cognitive impairment on the Mini-Mental Status Examination (score ≥ 24) were included in the study. Older adults with severe medical conditions, such as uncontrolled hypertension and acute pain, daily opioids use, or acute symptoms that interfered with data collection were excluded from the study. Although the participants were recruited in Korea and the USA simultaneously, they were not matched.

The sample size requirements for the structural equation modeling approach have diverse rules-of-thumb methods, such as 5–15 cases per measurements variable [21–23]. Given that we used nine measurement variables in this study, a range of 45–135 cases was needed in each of the two groups studied (Koreans and KAs). Hence, the sample size in this study was considered appropriate for building structural equation models.

### Measurements

#### Background characteristics

For this study, we retrieved data from the original study on: age, gender, perceived financial status (very hard to make a living/a little hard to make a living/not hard to make a living at all), the number of comorbidities, and the level of acculturation (for only older KAs) [11]. The level of acculturation was assessed by asking questions about preferred foods, customs, music, language, and close friendship on a 5-point Likert scale [24, 25]. The possible score range was from one to five, with higher scores reflecting greater acculturation.

#### Depressive symptoms

The Korean version of the Patient Health Questionnaire-9 was used to assess the level of depressive symptoms in older adults [26]. This self-administered measure, which consists of nine items, asked the participants how often they have experienced nine problems related to depressive symptoms in the last 2 weeks. For each item, participants could choose one of the four responses ranging from “not at all” (0) to “nearly every day” (3). The possible score range was 0–27. The higher the total score, the higher the level of depressive symptoms. The coefficients of Cronbach’s alpha were 0.92 in a previous study [26], and 0.86 for Koreans, and 0.80 for KAs in this study, respectively.

#### Sleep quality

Sleep quality of the participants was assessed by the Korean version of the Pittsburg Sleep Quality Index [27]. Among 24 items (19 self-administered and five reported by someone sleeping with the participant), this study used the19 items designed to be self-administered that asked that how often the participant had difficulties sleep in the past month. For each item, the participant could choose one of the four responses ranging from “not during the past month” to “three or more times a week”. The global score was yielded by summing the seven components generated by the combination of the items according to the scoring guideline. The possible score range was 0–21. The higher the global score, the worse the sleep quality. The Cronbach’s alpha coefficients were 0.84 in a previous study [27], and 0.72 for Koreans, and 0.79 for KAs in this study, respectively.

#### Chronic pain

The Korean version of the Brief Pain Inventory-Short Form was used to assess two domains including the pain severity and pain interference [28]. Four items for the pain severity (now, average, worst, least) and nine for the pain interference (e.g., general activity, mood, walking ability, ordinary work, interpersonal relation, sleep, enjoying life) asked the participant to choose from an 11-point scale ranging from “no pain” or “no interference” (0) to “pain as bad as you can imagine” or “interferes completely” (10). The total scores were calculated by summing all items in each domain in accordance with the scoring guideline [29]. The possible score range was 0–40 for the severity and 0–90 for the interference. The higher the score in each domain, the worse the pain or the more pain interferes with daily life, respectively. The study for the development of the Korean version reported the alpha coefficients were 0.85 for the pain severity and 0.93 for the pain interference [28]. In this study, the alpha coefficients were 0.90 for the pain severity and 0.92 for the pain interference in Koreans and 0.86 and 0.91, respectively, in KAs.

#### Quality of life

The Korean version of the Euro Quality of Life Questionnaire 5-Dimensional Classification-3L (EQ-5D) was used to assess quality of life [30]. This measure consists of five items including mobility, self-care, daily activities, pain/discomfort, and anxiety/depression using a three-level scale as calculating an EQ-5D value and one item for self-rated health status using a visual analog scale (EQ VAS). The possible score range was 0–1 for the EQ-5D value and 0–100 for the EQ VAS. The higher the scores, the better the quality of life. The study for the development of the Korean version reported acceptable validity [30]. In this study, the alpha coefficients were 0.81 for Koreans and 0.79 for KAs.

### Data collection

Data collection for the original study was carried out from November 2018 to March 2019 in Maryland, U.S., and Seoul, Korea. The participants were simultaneously recruited from a senior welfare center in Seoul, Korea, and from multiple sites, including Korean senior centers, Korean churches, and elderly day care centers, in Maryland, U.S. The recruitment places were selected to make the functional status of the participants in Korea and in the USA similar. Basically, the participants filled out the questionnaires by themselves; however, the researchers assisted the completion of the questionnaires when the participants want.

### Ethical consideration

The original study was approved by the institutional review boards (IRBs) at the Towson University in the U.S. (#1,806,036,244) and Seoul National University in Korea (1808/002-009). This secondary data analysis was also approved by the IRB of the Seoul National University (E2005/003-006). When the older adults showed a willingness to participate in the study, the researchers provided sufficient information on the study purpose, time for the survey, possible benefits and risks, and freedom of voluntarily withdrawal from the study without any disadvantage. Among those who understood the purpose of the study and wished to participate in it, the researchers screened the eligibility in accordance with the inclusion and exclusion criteria. Then, written informed consent from all participants was obtained.

### Data analysis

IBM SPSS version 23.0 and AMOS version 23.0 were used to analyze the data. First, all data were reviewed to check for the presence of missing values. Approximately 0.2% of the data were missing, so they were dealt with expectation–maximization. Background characteristics and all study variables were described by mean and standard deviation for continuous data and frequencies and percentage for categorical data. They were also compared by groups (Koreans vs. KAs) using independent t-tests or chi-square tests. In order to check the assumptions for a structural equation modeling analysis, variables including age, the number of comorbidities, depressive symptoms, sleep quality, perceived financial status, acculturation, pain, and quality of life confirmed no signs of multicollinearities using Pearson correlation analyses, and normality using skewness and kurtosis. Then, the measurement equivalence among two groups, which indicates comparability of measurement in the cross-national study [31, 32], was confirmed by multiple group confirmatory factor analysis.

The structural equation model in each group was using a maximum likelihood estimation and bootstrapping. The model fit was evaluated using multiple indices including relative *χ*^2^ below 3, goodness of fit index (GFI) above 0.90, normed fit index (NFI) above 0.90, comparative fit index (CFI) above 0.90, incremental fit index (IFI) above 0.90, and root mean squared error of approximation below 0.10 in each model [32, 33]. The parameter estimates in each structural equation models were analyzed using standardized regression weights (*β*), standard errors (S.E.), critical ratio (C.R.), and squared multiple correlations (SMC). In addition, the multifaceted elements, including biological (age, the number of comorbidities), psychological (depressive symptoms, sleep quality), and social (perceived financial status, acculturation) aspects on chronic pain and quality of life, were examined for direct, indirect, and total effects, which were reported by standard regression coefficients (*β*) and *p-*values. Acculturation was input in the model of KAs in this study.

## Results

### Background characteristics and descriptive statistics for all variables

The mean age of the Korean participants was 75.60 years, and the mean age of the of the KA participants was 78.33 years (Table 1). Most of the Korean participants perceived their financial status as a little hard to make a living (47.8%) or not hard to make a living (40.6%), and they had an average of 2.2 comorbidities. Meanwhile, more than a half of KAs perceived their financial status as not hard to make a living (59.1%), and they had an average of 2.68 comorbidities and the mean level of acculturation was 1.59.

**Table 1:** Background characteristics and variables among the participants (*n* = 270)

Characteristics	Koreans (*N* = 138)	Korean–Americans (*N* = 132)	*P*-value
	*M* ± SD or *N* (%)		
Age, years			
65–74	61 (44.2)	37 (28.0)	
75–84	65 (47.1)	71 (53.8)	
≥ 85	12 (8.7)	24 (18.2)	
	75.69 ± 6.53	78.33 ± 6.68	.001
Gender			
Male	61 (44.2)	24 (18.2)	< .001
Female	77 (55.8)	108 (81.8)	
Financial status			
Very hard to make a living	16 (11.6)	10 (7.6)	.010
A little hard to make a living	66 (47.8)	44 (33.3)	
Not hard to make a living	56 (40.6)	134 (59.1)	
The number of comorbidities (range 0–9)	2.20 ± 1.27	2.68 ± 1.87	.014
Level of acculturation (range 0–5)	–	1.59 ± 0.04	
*Variables*			
Depressive symptoms (range 0–27)	4.39 ± 4.85	5.55 ± 5.34	.064
Sleep quality (range 0–21)	6.54 ± 3.50	7.69 ± 4.64	.030
Chronic pain			
Severity (range 0–40)	12.23 ± 7.95	15.06 ± 8.72	.006
Interference (range 0–90)	17.28 ± 14.60	22.92 ± 18.13	.028
Quality of life			
EQ-5D value (range 0–1)	0.82 ± 0.12	0.75 ± 0.18	< .001
EQ VAS (range 0–100)	75.78 ± 16.89	65.62 ± 20.37	< .001

The mean depressive symptoms score was not significantly different between the two groups (4.39 for Koreans and 5.55 for KAs). The mean scores for the other variables were significantly different between the two groups: sleep quality (6.54 for Koreans, 7.69 for KAs), chronic pain severity (12.23 for Koreans, 15.06 for KAs), chronic pain interference (17.28 for Koreans, 22.92 for KAs), quality of life (0.82 for Koreans, 0.75 for KAs), and self-rated health status (75.78 for Koreans, 65.62 for KAs) (Table 1).

### Structural equation modeling in each group

**Fig. 2:**
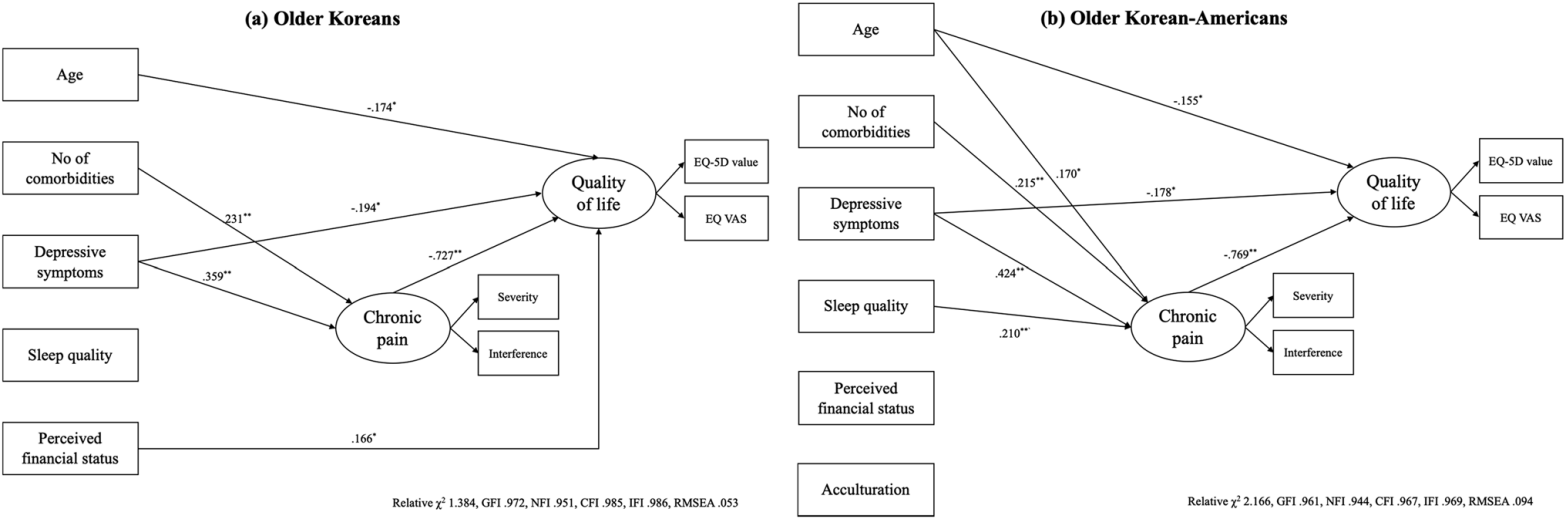
The results of the structural equation modeling

The results of the structural equation models in older Koreans and KAs with chronic pain appear in Fig. 2 and Table 2. As illustrated in Fig. 2, the fit indices of each model were satisfied with criterion (relative *χ*^2^ of 1.384, GFI of .972, NFI of .951, CFI of .985, IFI of .986, and RMSEA of .053 for Koreans; relative *χ*^2^ of 2.166, GFI of .961, NFI of .944, CFI of .967, IFI of .969, and RMSEA of .094 for KAs).

**Table 2:** Parameter estimates in each structural equation models (*n* = 270)

Exogenous	Endogenous	Koreans (*n* = 138)					Korean–Americans (*n* = 132)				
		*β*	S.E.	C.R.	*p*	SMC	*β*	S.E.	C.R.	*p*	SMC
Age	Chronic pain	.066	0.165	0.870	.384	.284	.170	0.196	2.348	.019*	.429
The number of comorbidities		.231	0.859	2.993	.003**		.215	0.698	2.986	.003**	
Depressive symptoms		.359	0.246	4.239	< .001**		.424	0.290	4.821	< .001**	
Sleep quality		.148	0.328	1.831	.067		.210	0.312	2.631	.009*	
Perceived financial status		.004	1.683	0.046	.963		.141	2.202	1.837	.066	
Acculturation		–	–	–	–		.060	0.528	0.838	.402	

Age	Quality of life	− .174	0.001	− 2.432	.015*	.887	− .155	0.001	− 2.308	.021*	.955
The number of comorbidities		− .088	0.006	− 1.165	.244		− .118	0.005	− 1.746	.081	
Depressive symptoms		− .194	0.002	− 2.217	.027*		− .178	0.002	− 2.041	.041*	
Sleep quality		.010	0.002	0.136	.892		.023	0.002	0.310	.756	
Perceived financial status		.166	0.010	2.245	.025*		− .039	0.018	− 0.521	.602	
Acculturation		–	–	–	–		.050	0.004	0.775	.438	
Chronic pain		− .727	0.001	− 6.425	< .001**		− .769	0.001	− 9.509	< .001**	

*β* standardized regression weights, *S.E.* standard errors, *C.R.* critical ratio, *SMC* squared multiple correlations

*p < .05

**p < .01

In the model for Koreans (Fig. 2 and Table 2), there were two significant paths to chronic pain: the number of comorbidities (*β* = .231, *p* = .003) and depressive symptoms (*β* = .359, *p* < .001). In addition, each path from age (*β* = − .174, *p* = .015), depressive symptoms (*β* = − .194, *p* = .027), perceived financial status (*β* = .166, *p* = .025), and chronic pain (*β* = − .727, *p* < .001) to quality of life in Koreans was significant.

In the model for KAs (Fig. 2 and Table 2), there were four significant paths to chronic pain: age (*β* = .170, *p* = .019), the number of comorbidities (*β* = .215, *p* = .003), depressive symptoms (*β* = .424, *p* < .001), and sleep quality (*β* = .210, *p* = .009). In addition, each path from age (*β* = − .155, *p* = .021), depressive symptoms (*β* = − .178, *p* = .041), and chronic pain (*β* = − .769, *p* < .001) to quality of life in KAs was significant.

Standardized direct, indirect, and total effects in each model are summarized in Tables 3 and 4. In the model for Koreans (Table 3), the number of comorbidities (*β* = .231, *p* = .034) and depressive symptoms (*β* = .359, *p* = .004) had direct and total effects on chronic pain. Among the variables that had total effects on quality of life, including age (*β* = − .222, *p* = .003), the number of comorbidities (*β* = − .256, *p* = .006), depressive symptoms (*β* = − .455, *p* = .004), and chronic pain (*β* = − .727, *p* = .002), the number of comorbidities and depressive symptoms indirectly affected quality of life through chronic pain even though they did not directly affect it.

**Table 3:** Direct, indirect, and total effects in the structural equation model for older Koreans with chronic pain (*n* = 138)

Exogenous	Endogenous	Standardized total effect		Standardized direct effect		Standardized indirect effect	
		*β*	*p*-value	*β*	*p*-value	*β*	*p*-value
Age	Chronic pain	.143	.456	.066	.456		
The number of comorbidities		.231	.034*	.231	.034*		
Depressive symptoms		.359	.004**	.359	.004**		
Sleep quality		.148	.135	.148	.135		
Perceived financial status		.004	.967	.004	.967		

Age	Quality of life	− .222	.003**	− .174	.010**	− .048	.432
The number of comorbidities		− .256	.006**	− .088	.185	− .168	.024*
Depressive symptoms		− .455	.004**	− .194	.165	− .261	.002**
Sleep quality		− .097	.337	.010	.994	− .108	.104
Perceived financial status		.163	.057	.166	.027*	− .003	.959
Chronic pain		− .727	.002**	− .727	.002**		

*p < .05

**p < .01

**Table 4:** Direct, indirect, and total effects in the structural equation model for older Korean–Americans with chronic pain (*n* = 132)

Exogenous	Endogenous	Standardized total effect		Standardized direct effect		Standardized indirect effect	
		*β*	*p*-value	*β*	*p*-value	*β*	*p*-value
Age	Chronic pain	.170	.011*	.170	.011*		
The number of comorbidities		.215	.014*	.215	.014*		
Depressive symptoms		.424	.006**	.424	.006**		
Sleep quality		.210	.041*	.210	.041*		
Perceived financial status		.141	.098	.141	.098		
Acculturation		.060	.346	.060	.346		

Age	Quality of life	− .285	.011*	− .155	.027*	− .130	.008**
The number of comorbidities		− .283	.018*	− .118	.181	− .165	.011*
Depressive symptoms		− .504	.004**	− .178	.118	− .326	.003**
Sleep quality		− .138	.207	.023	.785	− .161	.030*
Perceived financial status		− .148	.095	− .039	.609	− .109	.098
Acculturation		.004	.998	.050	.493	− .046	.346
Chronic pain		− .769	.002**	− .769	.002**		

*p < .05

**p < .01

In the model for KAs (Table 4), age (*β* = .170, *p* = .011), the number of comorbidities (*β* = .215, *p* = .014), depressive symptoms (*β* = .424, *p* = .006), and sleep quality (*β* = .210, *p* = .041) had direct and total effects on chronic pain. Among the variables that had total effects on quality of life, including age (*β* = − .285, *p* = .011), the number of comorbidities (*β* = − .283, *p* = .018), depressive symptoms (*β* = − .504, *p* = .004), and chronic pain (*β* = − .769, *p* = .002), the number of comorbidities and depressive symptoms only indirectly influenced quality of life through chronic pain, as in the model for KAs. Although sleep quality had an indirect effect on quality of life through chronic pain, the total effect was not significant.

## Discussion

This study presents potential factors for understanding chronic pain and improving quality of life by exploring relationships among multifaceted elements, chronic pain, and quality of life in older adults with chronic pain. Particularly, this study revealed that there were no statistically significant cultural effects on chronic pain and quality of life by comparing the pathways among older adults who had same ethnicity but living in different cultures. This lack of effect could be explained by the low level of acculturation among the older KAs, in that most of them still maintain a Korean lifestyle despite living in the USA. Several previous studies have also found a lower level of acculturation in older Korean immigrants compared with other Asian immigrants [34, 35]. In fact, additional analysis including acculturation levels for KAs in our structural equation model revealed no significant effects of acculturation on either chronic pain or quality of life (data not shown). These findings suggest that acculturation among elderly immigrants may not be a strong variant predicting health outcomes because they tend to have low level of acculturation in general. Nevertheless, assessing cultural effects on health may be important to better understand the Asian elderly immigrants’ health and healthcare use [19] because low acculturation level may suggest a lower likelihood of adjusting to the new culture.

The findings that the number of comorbidities and depressive symptoms had positive effects on chronic pain in both groups were in line with previous studies. Several studies have reported that the higher the number of comorbidities, the more severe the pain intensity and the longer the pain duration [11, 36–38]. This effect might be because chronic pain often results from other health problems, such as musculoskeletal disorders, which are prevalent in older adults [6, 7]. Regarding depressive symptoms, previous studies have reported that about 13% of older adults have depression and chronic pain simultaneously [12, 39] and that older adults with depression are more vulnerable to chronic pain than those without depression [40, 41]. Although reasons for the coexistence of depression and chronic pain remain unclear, the evidence on claiming neuroinflammation in both diseases has been supported [42]. Multimorbidity and depressive symptoms are believed to play critical roles in chronic pain, especially in older Koreans, given that approximately 73% of older Koreans have at least two chronic diseases, with osteo/rheumatoid arthritis and low back pain/sciatica among the most prevalent chronic diseases, and that 21% suffer from depression [1].

The finding that age affected chronic pain in the model for older KAs is concordant with previous studies on chronic pain, which have commonly reported that age is one of the risk factors of chronic pain [10, 42] and that chronic pain prevalence tends to increase as age advances [2, 43]. However, this finding was not supported in the model for older Koreans in this study. This discrepancy in the relationship between chronic pain and age in the two groups might be related to the age differences in the older Korean and older KAs in our study. Our Korean sample included a relatively younger group than our KA sample, although the age ranges were similar. Hence, it can be inferred that the relationship between age and chronic pain is more clearly indicated by the inclusion of older adults aged 75 or over who have high pain prevalence and strong pain intensity [2, 44].

Both models for older Koreans and KAs with chronic pain showed that age, the number of comorbidities, depressive symptoms, and chronic pain had significant effects on quality of life, and these findings are consistent with previous studies [8, 41, 45]. Among these factors, chronic pain had the strongest effect on quality of life in both groups. As we pointed out earlier, chronic pain in older adults hinders functional independence in everyday activities, limits social interactions, and hampers leisure activities [46, 47]. Health care-related costs to manage chronic pain can also be financially burdensome [1, 5]. For those reasons, chronic pain may negatively influence quality of life in older Koreans and KAs in this study.

Perceived financial status had a positive direct effect on quality of life only in the older Koreans, whereas sleep quality had a negative indirect effect on quality of life through chronic pain only in the older KAs. A reason for this discrepancy between the two groups may be differences in social security benefits between the two locations that affect financial status. Most of the KAs who participated in this study were receiving Social Security Medicare Program benefits, and they were attending adult daycare centers where they could receive diverse benefits, including meals and health care services. For Korean elders, health insurance is covered by the government, but daily costs for living are relatively higher than in the USA, even in the elderly. The perceived financial status finding agrees with previous studies that reported a significant positive association between financial status and quality of life in older Koreans [48, 49]. That is, the finding on perceived financial status could be supported by the fact that most of the older Koreans in this study experienced more financial difficulties than the older KAs. Regarding sleep quality, the discrepancy between the two groups may be associated with more advanced age of the in KAs in the sample. Aging is associated with sleep quality [50], and poor sleep quality had a significant impact on chronic pain, as well as quality of life, in KAs. Previous studies identified poor sleep quality as leading to chronic pain by increasing pain sensitivity and, in turn, reducing quality of life [51, 52]. As in the previous studies, greater sleep disturbance in older KAs might negatively affect chronic pain and eventually result in poor quality of life. However, since both perceived financial status in older Koreans and sleep quality in older KAs did not have total effects on quality of life, the findings should be interpreted with a caution.

This study had some potential limitations. First, some variables, such as the number of comorbidities, depressive symptoms, sleep quality, chronic pain, and quality of life, are interrelated so that the associations could be bidirectional [42, 53]. However, we did not consider the bidirectional relationships because this study aimed to explore multifaceted elements affecting pain and quality of life. Second, as a secondary data analysis, this study used prearranged data. Although major variables affecting chronic pain and quality of life were included in the analyses, this study could not address additional information, such as pain management and immigration status. Lastly, the KAs included in this study were recruited from a region in the USA where a relatively large group of Koreans are living (Korean authentic foods and markets are available, and native language is commonly used), which may have influenced acculturation; thus, generalization of the results is limited.

## Conclusion

This study showed that multifaceted elements, including biological (age, the number of comorbidities), psychological (depressive symptoms, sleep quality), and social (perceived financial status) aspects, affect chronic pain and quality of life in older adults with chronic pain and that there were no cultural effects on chronic pain and quality of life in older adults who had the same ethnicity, but were living in different cultures. Some suggestions can be made based on the findings of this study. First, healthcare providers who come across older adults with chronic pain should examine affecting factors and relationships from various standpoints. In particular, when targeting older immigrants, healthcare providers should carefully assess older immigrants’ acculturation level using a valid measurement and consider integrating the acculturation level into an intervention program for alleviating chronic pain and improving quality of life. Further studies are needed to examine more comprehensive variables affecting quality of life in older immigrants with chronic pain. In addition, further studies are needed that use multiple measurements, such as a combination of direct (e.g., actigraphy) and indirect (e.g., sleep questionnaire) measurements, to provide a more accurate and extensive view of quality of life.

## Data Availability

Due to the nature of this research, participants for this study did not agree for their data to be shared publicly, so supporting data are not available.
